# Supplementary material for: A zebrafish screen reveals Renin-angiotensin system inhibitors as neuroprotective via mitochondrial restoration in dopamine neurons
Source: eLife. 2021 Sep 22;10:e69795. doi: 10.7554/eLife.69795 (PMC8457844; doi:10.7554/eLife.69795)

Danio rerio (zebrafish)
nuclear target - 10.7 kb
FWD 5’ AGAGCGCGATTGCTGGATTCAC 3’
REV 5’ GTCCTTGCAGGTTGGCAAATGG 3’

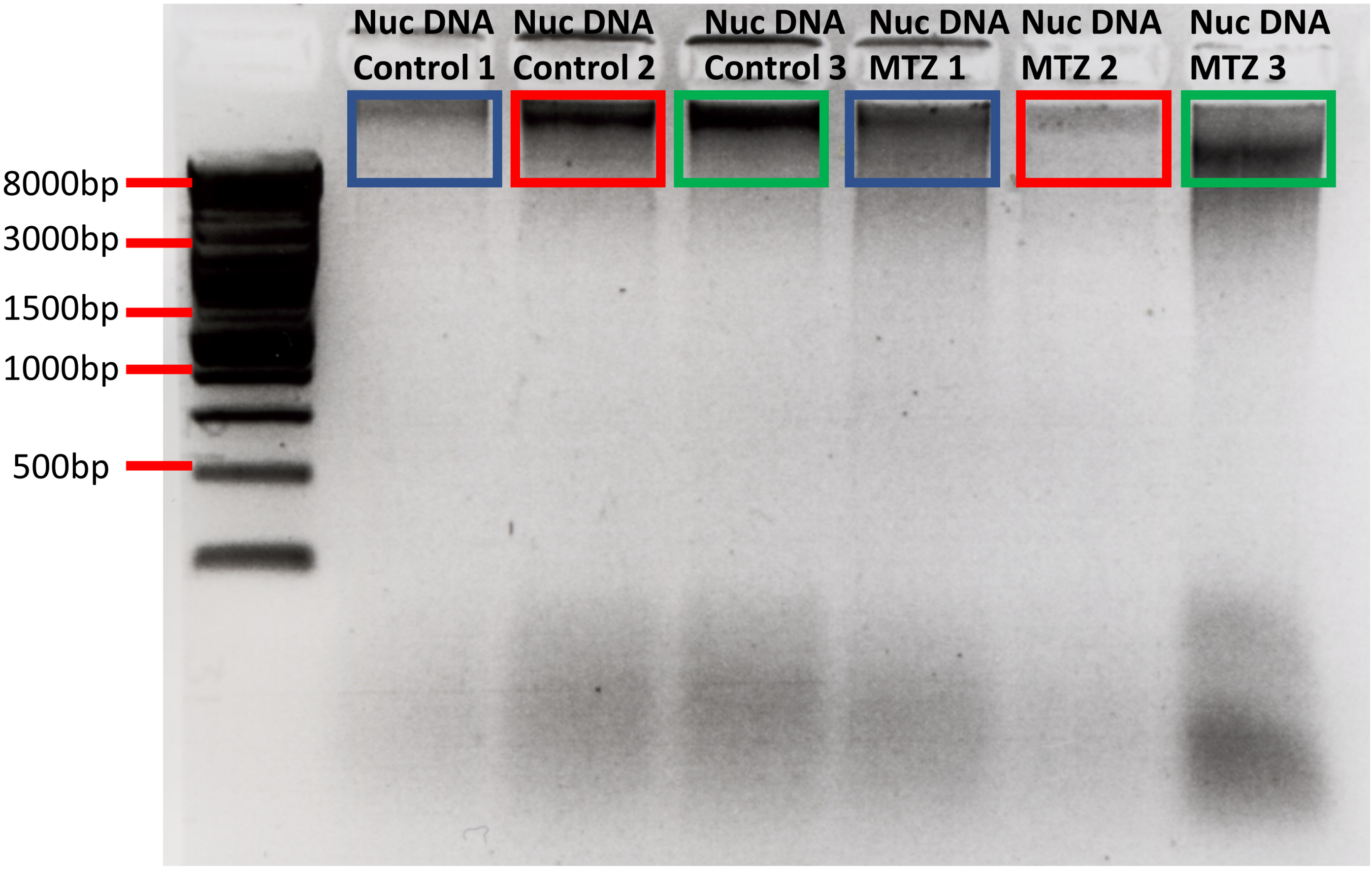

Supplement: Figure 1—source data 3. [file elife-69795-fig1-data3.pdf]
