# Supplementary figures and images for: A zebrafish screen reveals Renin-angiotensin system inhibitors as neuroprotective via mitochondrial restoration in dopamine neurons
Source: eLife. 2021 Sep 22;10:e69795. doi: 10.7554/eLife.69795 (PMC8457844; doi:10.7554/eLife.69795)

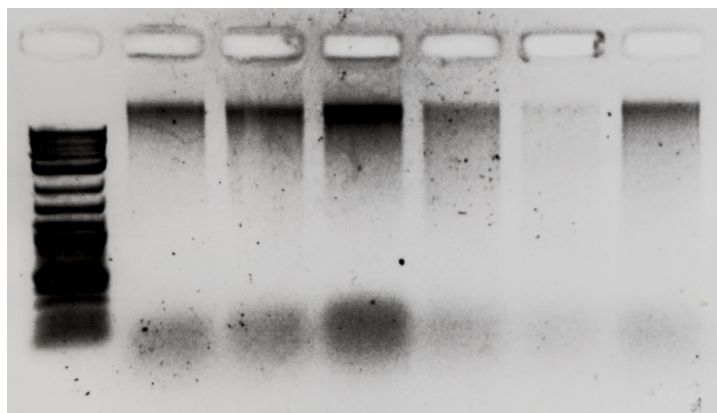

Supplement: Figure 1—source data 4. [file elife-69795-fig1-data4.pdf]

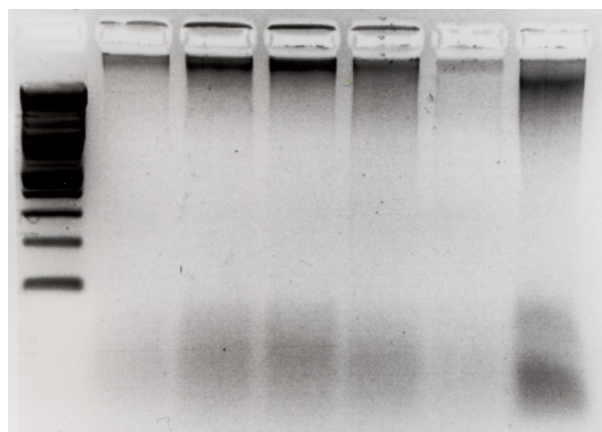

Supplement: Figure 1—source data 5. [file elife-69795-fig1-data5.pdf]

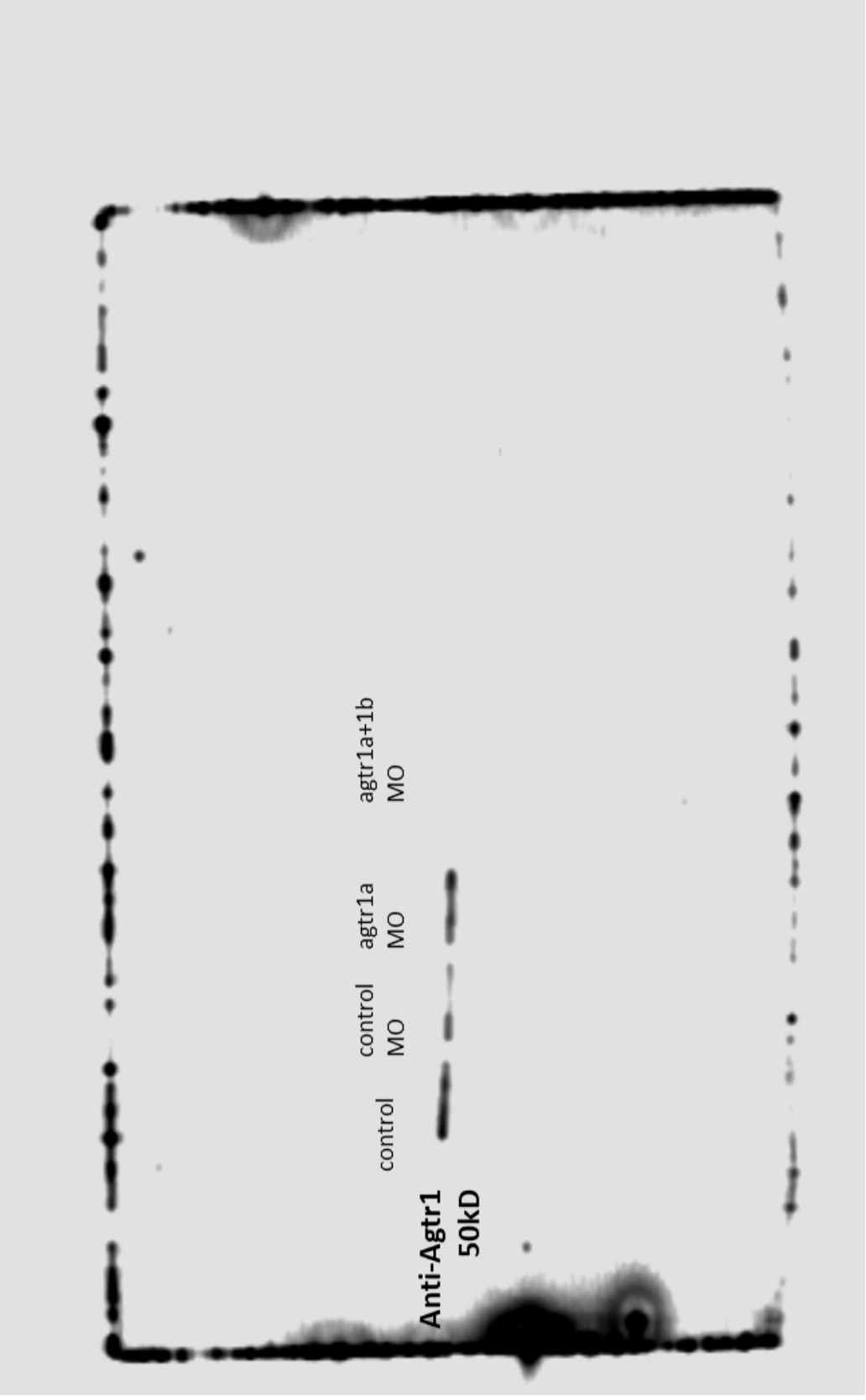

Supplement: Figure 2—figure supplement 3—source data 1. [file elife-69795-fig2-figsupp3-data1.pdf]

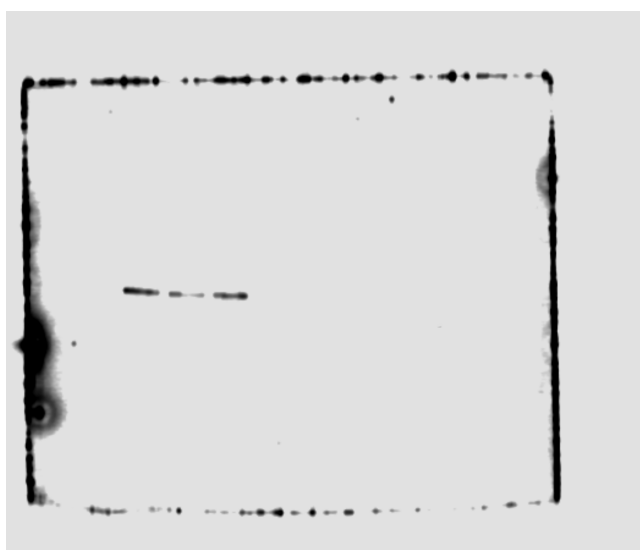

Supplement: Figure 2—figure supplement 3—source data 3. [file elife-69795-fig2-figsupp3-data3.pdf]

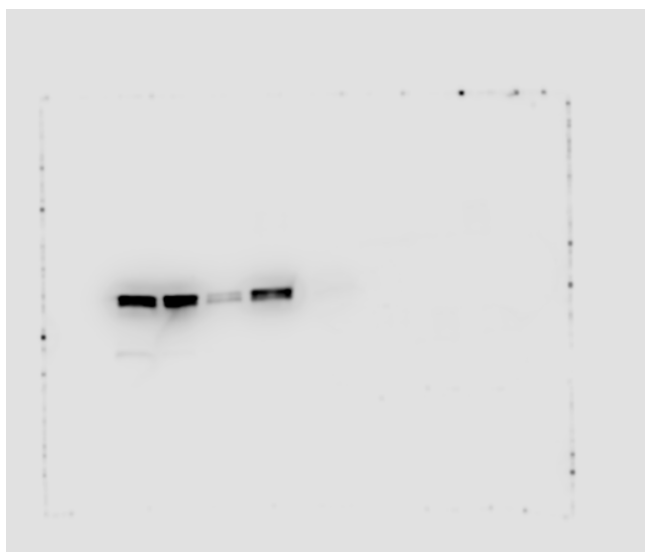

Supplement: Figure 2—figure supplement 3—source data 4. [file elife-69795-fig2-figsupp3-data4.pdf]
