# Supplementary material for: A zebrafish screen reveals Renin-angiotensin system inhibitors as neuroprotective via mitochondrial restoration in dopamine neurons
Source: eLife. 2021 Sep 22;10:e69795. doi: 10.7554/eLife.69795 (PMC8457844; doi:10.7554/eLife.69795)
Supplement: Figure 2—figure supplement 3—source data 2. [file elife-69795-fig2-figsupp3-data2.pdf]

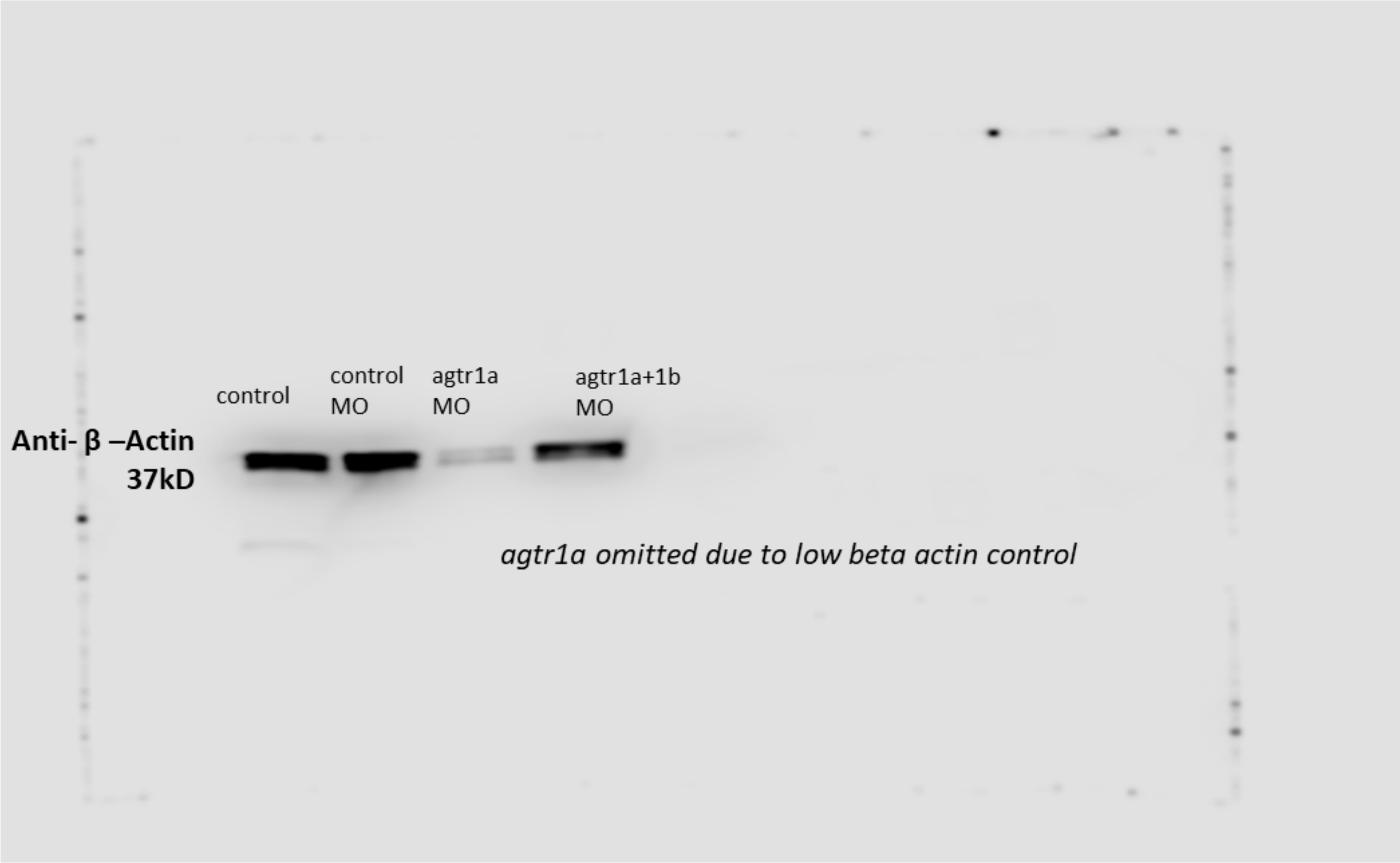
control
control MO
agtr1a MO
agtr1a+1b MO
Anti- β –Actin
37kD
agtr1a omitted due to low beta actin control
