# Supplementary material for: A zebrafish screen reveals Renin-angiotensin system inhibitors as neuroprotective via mitochondrial restoration in dopamine neurons
Source: eLife. 2021 Sep 22;10:e69795. doi: 10.7554/eLife.69795 (PMC8457844; doi:10.7554/eLife.69795)
Supplement: Figure 3—source data 2. [file elife-69795-fig3-data2.pdf]

Experiment Information

Name	Jeffrey 12/4/2018 2:08:31 PM
Date	12/4/2018 2:08:31 PM
Model Type	LE-SH800SZFCPL
Serial Number	1800024
Investigator	
Operator	
Memo	

Sample Group Information

Name	Sample Group - 1
Species	
Cell Type	
Memo	

Measurement Settings

Parameter Settings

	Marker	Fluorochrome	Acquisition Select Area	Height	Width
FSC	---	---	✓	✓	✓
BSC	---	---	✓		
FL1		Brilliant Violet 421			
FL2		EGFP	✓		
FL3		mCherry	✓		
FL4		APC			
FL5		PerCP-Cy5.5			
FL6		live/dead	✓		

Compensation Settings

Spillover Matrix (%)

Detector

Fluorochrome	EGFP	mCherry	live/dead
EGFP	100.00	4.66	0.00
mCherry	26.06	100.00	0.00
live/dead	0.00	0.00	100.00

Negative Value

Detector

	EGFP	mCherry	live/dead
Area	0	0	0
Height	0	0	0

Tube Information

Name	DM MTZ$~$IsNew
Sample ID1	
Sample ID2	
Sample ID3	
Sample ID4	

Basic Information

Description	
Model Type	LE-SH800SZFCPL
Serial Number	1800024
Chip Type	Sorting Chip
Chip ID	00040347-0000-7027
Nozzle Size	100 µm

Sample Stop Condition

None

Recording Setting

Type	Gated Event Count
Target Gate	Singlets
Value	30,000

Instrument Setting

Laser

405nm	Off
488nm	On
561nm	On
638nm	On

Threshold

Channel	FSC
Value	2.50%

Sensor Gain

FSC	16	BSC	42.5%
FL1	40.0%	FL2	40.0%
FL3	40.0%	FL4	40.0%
FL5	40.0%	FL6	42.0%

Sample Pressure 8

AD Advanced Setting

Forward Window Extension	50
Back Window Extension	

Auto Parameters

Droplet Clock	23,100	Hz
Droplet Drive	31.09	
Sort Delay	20	
Sort Phase	213	deg
Charge	50.0	%
Deflection Left	1,040	
Deflection Right	1,088	
Enabled Control Breakoff	☑	

Sorting Setting

Sorting Method 2 Way Tubes

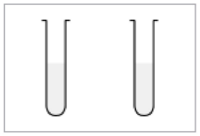

Collection Tube	Sort Gate	Sort Mode	Cell Size	Stop Count
Left	H	Purity	Large Cell	0
Right	G	Purity	Large Cell	0

Worksheet

All Events

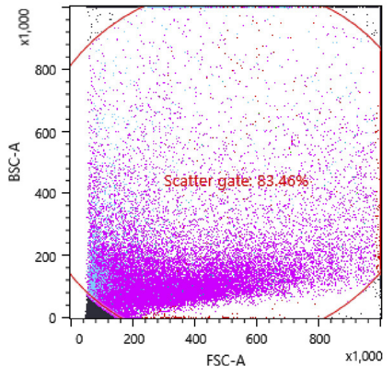

Scatter gate

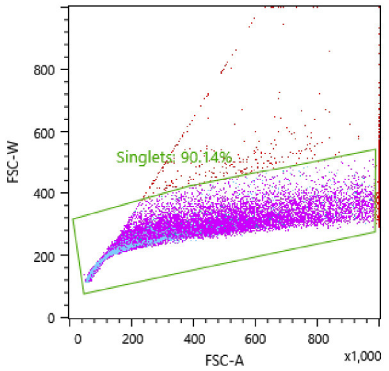

Gates and Statistics

Name	Events	%Parent	%Total
All Events	39,877	0.00%	100.00%
Scatter gate	33,280	83.46%	83.46%
Singlets	30,000	90.14%	75.23%
F	29,975	99.92%	75.17%
G	109	0.36%	0.27%
H	1,361	4.54%	3.41%

Singlets

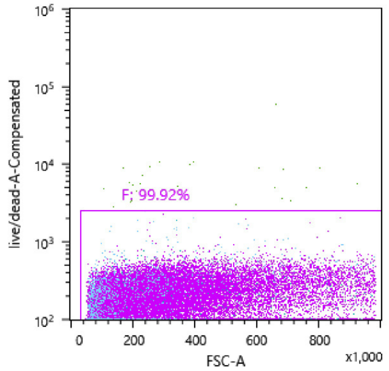

F

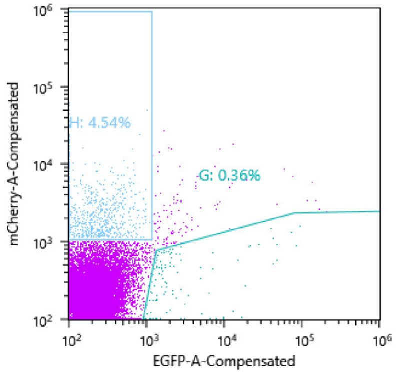

All Events

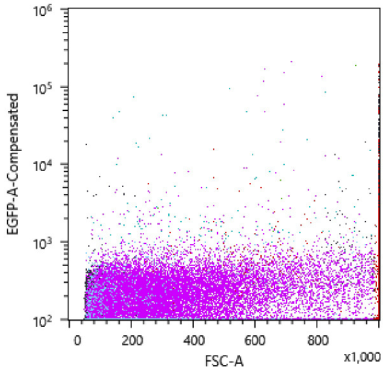

Gates and Statistics

Name	Events	%Parent	%Total
All Events	39,877	0.00%	100.00%
Scatter gate	33,280	83.46%	83.46%
Singlets	30,000	90.14%	75.23%
F	29,975	99.92%	75.17%
G	109	0.36%	0.27%
H	1,361	4.54%	3.41%

Recording Result

Total Record Count:	39,877
Start Time:	12/4/2018 2:26:29 PM
End Time:	12/4/2018 2:26:47 PM

Sorting Result

Sorting Method 2 Way Tubes

Start Time 12/4/2018 2:25:01 PM

End Time 12/4/2018 2:31:00 PM

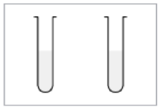

Collection Tube	Sort Gate	Sort Mode	Elapsed Time	Total Event	Target Ratio	Sorted Count	Sort Rate	Sort Efficiency	Aborted Count	Abort Rate
Left	H	Purity	00:05:59	811,953	3.84%	25,709	71.52eps	82.53%	5,442	15.14eps
Right	G	Purity	00:05:59	811,953	0.24%	1,631	4.54eps	82.83%	338	0.94eps

Experiment Information

Name	Jeffrey 12/4/2018 2:08:31 PM
Date	12/4/2018 2:08:31 PM
Model Type	LE-SH800SZFCPL
Serial Number	1800024
Investigator	
Operator	
Memo	

Sample Group Information

Name	Sample Group - 1
Species	
Cell Type	
Memo	

Measurement Settings

Parameter Settings

	Marker	Fluorochrome	Acquisition Select Area	Height	Width
FSC	---	---	✓	✓	✓
BSC	---	---	✓		
FL1		Brilliant Violet 421			
FL2		EGFP	✓		
FL3		mCherry	✓		
FL4		APC			
FL5		PerCP-Cy5.5			
FL6		live/dead	✓		

Compensation Settings

Spillover Matrix (%)

Fluorochrome \ Detector	EGFP	mCherry	live/dead
EGFP	100.00	4.66	0.00
mCherry	26.06	100.00	0.00
live/dead	0.00	0.00	100.00

Negative Value

Detector	EGFP	mCherry	live/dead
Area	0	0	0
Height	0	0	0

Tube Information

Name	DM
Sample ID1	
Sample ID2	
Sample ID3	
Sample ID4	

Basic Information

Description	
Model Type	LE-SH800SZFCPL
Serial Number	1800024
Chip Type	Sorting Chip
Chip ID	00040347-0000-7027
Nozzle Size	100 µm

Sample Stop Condition

None

Recording Setting

Type	Gated Event Count
Target Gate	Singlets
Value	10,000

Instrument Setting

Laser

405nm	Off
488nm	On
561nm	On
638nm	On

Threshold

Channel	FSC
Value	4.00%

Sensor Gain

FSC	16	BSC	42.5%
FL1	40.0%	FL2	40.0%
FL3	40.0%	FL4	40.0%
FL5	40.0%	FL6	42.0%

Sample Pressure 8

AD Advanced Setting

Forward Window Extension	50
Back Window Extension	

Auto Parameters

Droplet Clock	23,100	Hz
Droplet Drive	30.90	
Sort Delay	20	
Sort Phase	213	deg
Charge	50.0	%
Deflection Left	1,160	
Deflection Right	968	
Enabled Control Breakoff	✔	

Sorting Setting

Sorting Method 2 Way Tubes

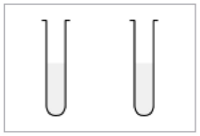

Collection Tube	Sort Gate	Sort Mode	Cell Size	Stop Count
Left	H	Purity	Regular Cell	0
Right	G	Purity	Regular Cell	0

Worksheet

All Events

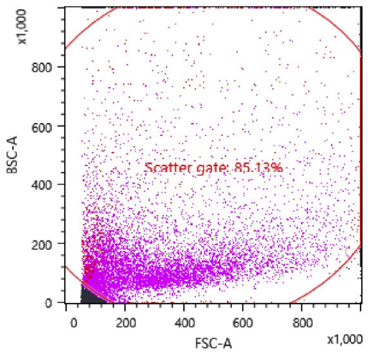

Scatter gate

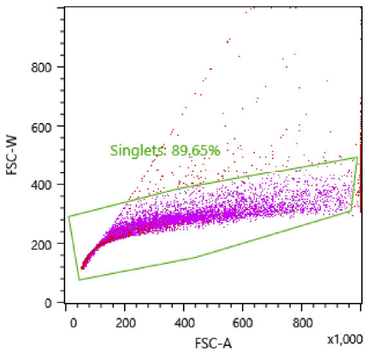

Gates and Statistics

Name	Events	%Parent	%Total
All Events	13,103	0.00%	100.00%
Scatter gate	11,154	85.13%	85.13%
Singlets	10,000	89.65%	76.32%
F	9,987	99.87%	76.22%
G	74	0.74%	0.56%
H	510	5.11%	3.89%

Singlets

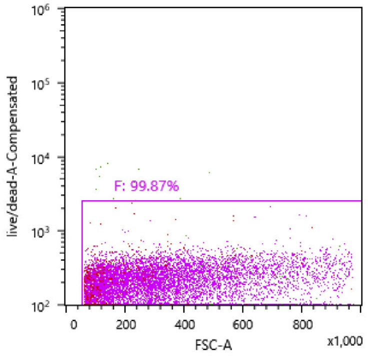

F

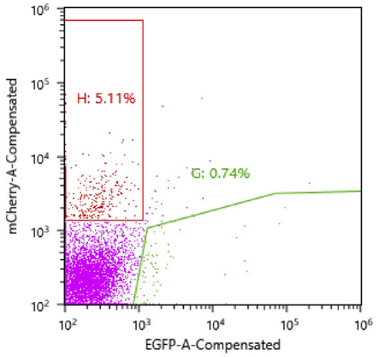

All Events

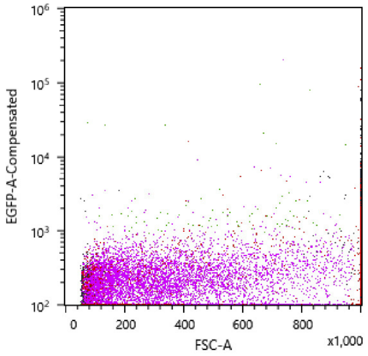

Gates and Statistics

Name	Events	%Parent	%Total
All Events	13,103	0.00%	100.00%
Scatter gate	11,154	85.13%	85.13%
Singlets	10,000	89.65%	76.32%
F	9,987	99.87%	76.22%
G	74	0.74%	0.56%
H	510	5.11%	3.89%

Recording Result

Total Record Count:	13,103
Start Time:	12/4/2018 2:15:46 PM
End Time:	12/4/2018 2:15:57 PM

Sorting Result

Sorting Method	2 Way Tubes
Start Time	12/4/2018 2:14:53 PM
End Time	12/4/2018 2:21:25 PM

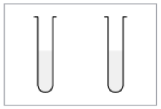

Collection Tube	Sort Gate	Sort Mode	Elapsed Time	Total Event	Target Ratio	Sorted Count	Sort Rate	Sort Efficiency	Aborted Count	Abort Rate
Left	H	Purity	00:06:32	805,596	3.15%	21,074	53.75eps	83.02%	4,309	10.99eps
Right	G	Purity	00:06:32	805,596	0.48%	3,203	8.17eps	82.66%	672	1.71eps

Experiment Information

Name	Jeffrey 12/4/2018 2:08:31 PM
Date	12/4/2018 2:08:31 PM
Model Type	LE-SH800SZFCPL
Serial Number	1800024
Investigator	
Operator	
Memo	

Sample Group Information

Name	Sample Group - 1
Species	
Cell Type	
Memo	

Measurement Settings

Parameter Settings

	Marker	Fluorochrome	Acquisition Select Area	Height	Width
FSC	---	---	✓	✓	✓
BSC	---	---	✓		
FL1		Brilliant Violet 421			
FL2		EGFP	✓		
FL3		mCherry	✓		
FL4		APC			
FL5		PerCP-Cy5.5			
FL6		live/dead	✓		

Compensation Settings

Spillover Matrix (%)

Detector

Fluorochrome	EGFP	mCherry	live/dead
EGFP	100.00	4.66	0.00
mCherry	26.06	100.00	0.00
live/dead	0.00	0.00	100.00

Negative Value

Detector

	EGFP	mCherry	live/dead
Area	0	0	0
Height	0	0	0

Tube Information

Name	Homo - 1$~$IsNew
Sample ID1	
Sample ID2	
Sample ID3	
Sample ID4	

Basic Information

Description	
Model Type	LE-SH800SZFCPL
Serial Number	1800024
Chip Type	Sorting Chip
Chip ID	00040347-0000-7027
Nozzle Size	100 µm

Sample Stop Condition

None

Recording Setting

Type	Gated Event Count
Target Gate	Singlets
Value	30,000

Instrument Setting

Laser

405nm	Off
488nm	On
561nm	On
638nm	On

Threshold

Channel	FSC
Value	2.50%

Sensor Gain

FSC	16	BSC	42.5%
FL1	40.0%	FL2	40.0%
FL3	40.0%	FL4	40.0%
FL5	40.0%	FL6	42.0%

Sample Pressure 8

AD Advanced Setting

Forward Window Extension	50
Back Window Extension	

Auto Parameters

Droplet Clock	23,100	Hz
Droplet Drive	31.31	
Sort Delay	20	
Sort Phase	213	deg
Charge	50.0	%
Deflection Left	1,040	
Deflection Right	1,088	
Enabled Control Breakoff	☑	

Sorting Setting

Sorting Method 2 Way Tubes

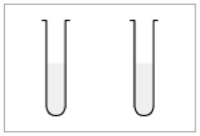

Collection Tube	Sort Gate	Sort Mode	Cell Size	Stop Count
Left	H	Purity	Large Cell	0
Right	G	Purity	Large Cell	0

Worksheet

All Events

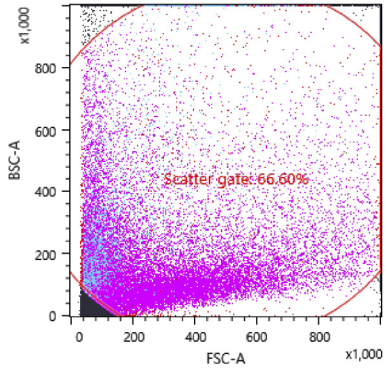

Scatter gate

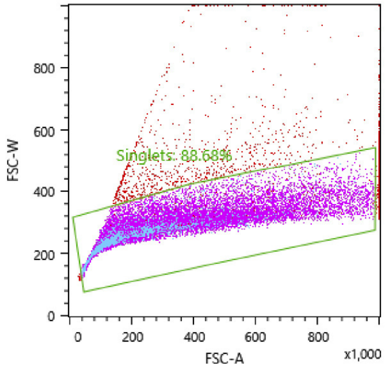

Gates and Statistics

Name	Events	%Parent	%Total
All Events	50,792	0.00%	100.00%
Scatter gate	33,828	66.60%	66.60%
Singlets	30,000	88.68%	59.06%
F	29,994	99.98%	59.05%
G	99	0.33%	0.19%
H	2,057	6.86%	4.05%

Singlets

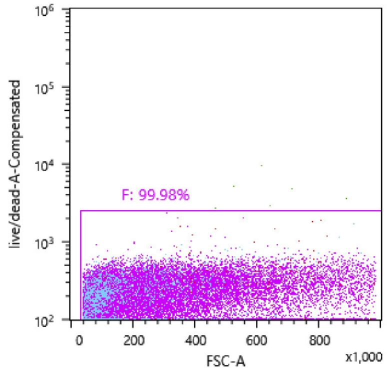

F

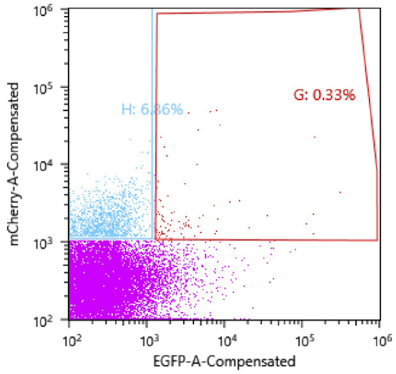

All Events

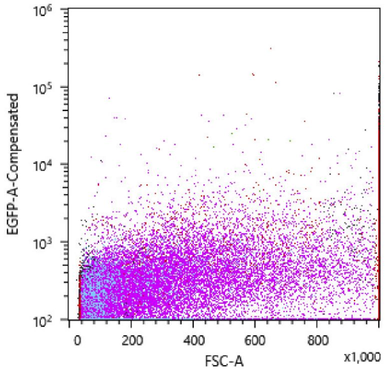

H

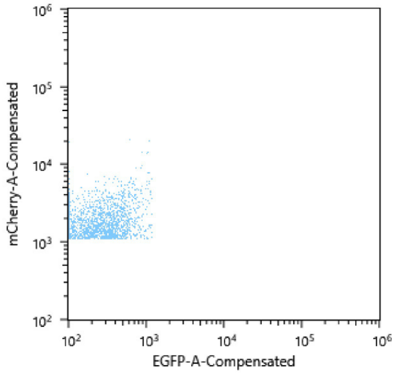

Gates and Statistics

Name	Events	%Parent	%Total
All Events	50,792	0.00%	100.00%
Scatter gate	33,828	66.60%	66.60%
Singlets	30,000	88.68%	59.06%
F	29,994	99.98%	59.05%
G	99	0.33%	0.19%
H	2,057	6.86%	4.05%

Recording Result

Total Record Count:	50,792
Start Time:	12/4/2018 2:41:14 PM
End Time:	12/4/2018 2:41:23 PM

Sorting Result

Sorting Method 2 Way Tubes

Start Time 12/4/2018 2:38:03 PM

End Time 12/4/2018 2:46:43 PM

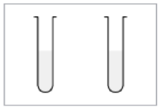

Collection Tube	Sort Gate	Sort Mode	Elapsed Time	Total Event	Target Ratio	Sorted Count	Sort Rate	Sort Efficiency	Aborted Count	Abort Rate
Left	H	Purity	00:08:39	3,205,799	3.85%	75,409	145.03eps	61.17%	47,877	92.08eps
Right	G	Purity	00:08:39	3,205,799	0.21%	3,964	7.62eps	58.33%	2,832	5.45eps

Experiment Information

Name	Jeffrey 12/4/2018 2:08:31 PM
Date	12/4/2018 2:08:31 PM
Model Type	LE-SH800SZFCPL
Serial Number	1800024
Investigator	
Operator	
Memo	

Sample Group Information

Name	Sample Group - 1
Species	
Cell Type	
Memo	

Measurement Settings

Parameter Settings

	Marker	Fluorochrome	Acquisition Select Area	Acquisition Select Height	Acquisition Select Width
FSC	---	---	✓	✓	✓
BSC	---	---	✓		
FL1		Brilliant Violet 421			
FL2		EGFP	✓		
FL3		mCherry	✓		
FL4		APC			
FL5		PerCP-Cy5.5			
FL6		live/dead	✓		

Compensation Settings

Spillover Matrix (%)

Fluorochrome \ Detector	EGFP	mCherry	live/dead
EGFP	100.00	4.66	0.00
mCherry	26.06	100.00	0.00
live/dead	0.00	0.00	100.00

Negative Value

Detector	EGFP	mCherry	live/dead
Area	0	0	0
Height	0	0	0

Tube Information

Name	Homo - MTZ$~$IsNew
Sample ID1	
Sample ID2	
Sample ID3	
Sample ID4	

Basic Information

Description	
Model Type	LE-SH800SZFCPL
Serial Number	1800024
Chip Type	Sorting Chip
Chip ID	00040347-0000-7027
Nozzle Size	100 µm

Sample Stop Condition

None

Recording Setting

Type	Gated Event Count
Target Gate	Singlets
Value	30,000

Instrument Setting

Laser

405nm	Off
488nm	On
561nm	On
638nm	On

Threshold

Channel	FSC
Value	2.50%

Sensor Gain

FSC	16	BSC	39.5%
FL1	40.0%	FL2	40.0%
FL3	40.0%	FL4	40.0%
FL5	40.0%	FL6	42.0%

Sample Pressure 8

AD Advanced Setting

Forward Window Extension	50
Back Window Extension	

Auto Parameters

Droplet Clock	23,100	Hz
Droplet Drive	31.48	
Sort Delay	20	
Sort Phase	213	deg
Charge	50.0	%
Deflection Left	1,040	
Deflection Right	1,088	
Enabled Control Breakoff	✔	

Sorting Setting

Sorting Method 2 Way Tubes

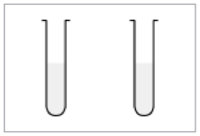

Collection Tube	Sort Gate	Sort Mode	Cell Size	Stop Count
Left	H	Purity	Large Cell	0
Right	G	Purity	Large Cell	0

Worksheet

All Events

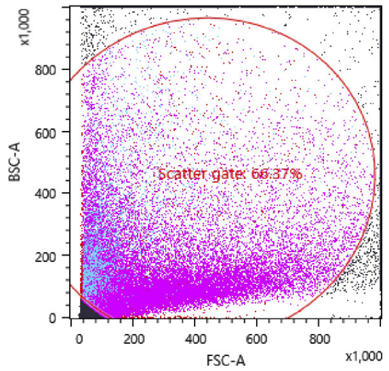

Scatter gate

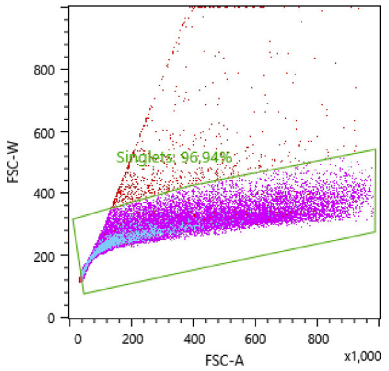

Gates and Statistics

Name	Events	%Parent	%Total
All Events	58,660	0.00%	100.00%
Scatter gate	38,935	66.37%	66.37%
Singlets	37,744	96.94%	64.34%
F	37,742	99.99%	64.34%
G	97	0.26%	0.17%
H	2,405	6.37%	4.10%

Singlets

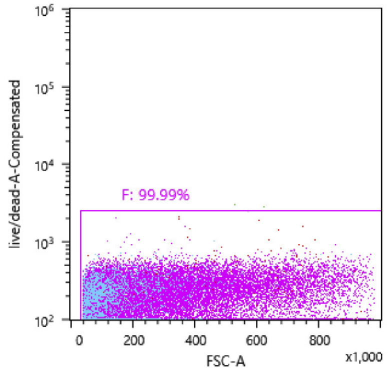

F

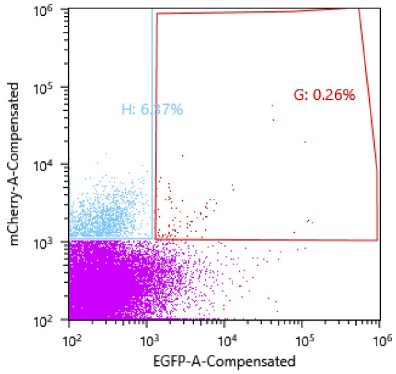

All Events

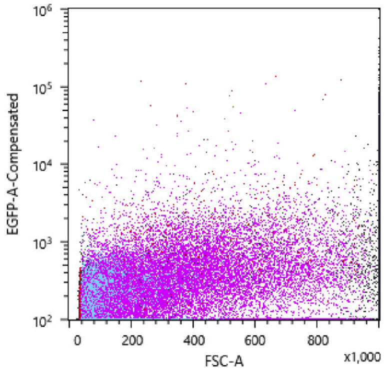

H

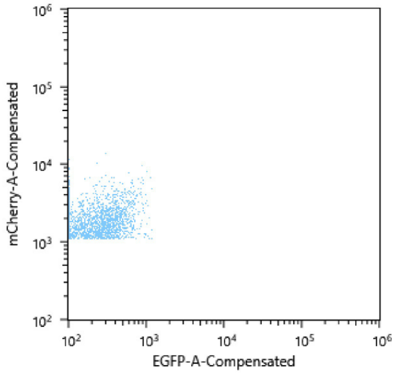

Gates and Statistics

Name	Events	%Parent	%Total
All Events	58,660	0.00%	100.00%
Scatter gate	38,935	66.37%	66.37%
Singlets	37,744	96.94%	64.34%
F	37,742	99.99%	64.34%
G	97	0.26%	0.17%
H	2,405	6.37%	4.10%

Recording Result

Total Record Count:	58,660
Start Time:	12/4/2018 2:50:08 PM
End Time:	12/4/2018 2:50:19 PM

Sorting Result

Sorting Method	2 Way Tubes
Start Time	12/4/2018 2:49:05 PM
End Time	12/4/2018 2:56:31 PM

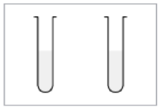

Collection Tube	Sort Gate	Sort Mode	Elapsed Time	Total Event	Target Ratio	Sorted Count	Sort Rate	Sort Efficiency	Aborted Count	Abort Rate
Left	H	Purity	00:07:26	2,468,291	4.43%	72,067	161.42eps	65.91%	37,277	83.50eps
Right	G	Purity	00:07:26	2,468,291	0.24%	3,909	8.76eps	65.15%	2,091	4.68eps

Experiment Information

Name	Jeffrey 12/4/2018 2:08:31 PM
Date	12/4/2018 2:08:31 PM
Model Type	LE-SH800SZFCPL
Serial Number	1800024
Investigator	
Operator	
Memo	

Sample Group Information

Name	Sample Group - 1
Species	
Cell Type	
Memo	

Measurement Settings

Parameter Settings

	Marker	Fluorochrome	Acquisition Select Area	Height	Width
FSC	---	---	✓	✓	✓
BSC	---	---	✓		
FL1		Brilliant Violet 421			
FL2		EGFP	✓		
FL3		mCherry	✓		
FL4		APC			
FL5		PerCP-Cy5.5			
FL6		live/dead	✓		

Compensation Settings

Spillover Matrix (%)

Fluorochrome \ Detector	EGFP	mCherry	live/dead
EGFP	100.00	4.66	0.00
mCherry	26.06	100.00	0.00
live/dead	0.00	0.00	100.00

Negative Value

Detector	EGFP	mCherry	live/dead
Area	0	0	0
Height	0	0	0

Tube Information

Name	Homo$~$IsNew
Sample ID1	
Sample ID2	
Sample ID3	
Sample ID4	

Basic Information

Description	
Model Type	LE-SH800SZFCPL
Serial Number	1800024
Chip Type	Sorting Chip
Chip ID	00040347-0000-7027
Nozzle Size	100 µm

Sample Stop Condition

None

Recording Setting

Type	Gated Event Count
Target Gate	Singlets
Value	30,000

Instrument Setting

Laser

405nm	Off
488nm	On
561nm	On
638nm	On

Threshold

Channel	FSC
Value	2.50%

Sensor Gain

FSC	16	BSC	42.5%
FL1	40.0%	FL2	40.0%
FL3	40.0%	FL4	40.0%
FL5	40.0%	FL6	42.0%

Sample Pressure 8

AD Advanced Setting

Forward Window Extension	50
Back Window Extension	

Auto Parameters

Droplet Clock	23,100	Hz
Droplet Drive	31.28	
Sort Delay	20	
Sort Phase	213	deg
Charge	50.0	%
Deflection Left	1,040	
Deflection Right	1,088	
Enabled Control Breakoff	☑	

Sorting Setting

Sorting Method 2 Way Tubes

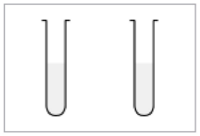

Collection Tube	Sort Gate	Sort Mode	Cell Size	Stop Count
Left	H	Purity	Large Cell	0
Right	G	Purity	Large Cell	0

Worksheet

All Events

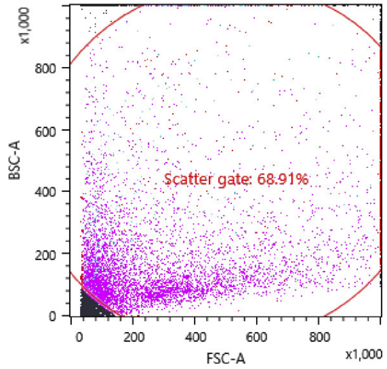

Scatter gate

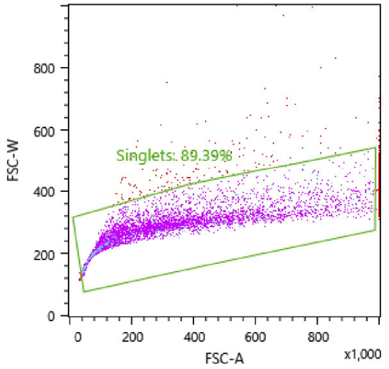

Gates and Statistics

Name	Events	%Parent	%Total
All Events	10,000	0.00%	100.00%
Scatter gate	6,891	68.91%	68.91%
Singlets	6,160	89.39%	61.60%
F	6,160	100.00%	61.60%
G	19	0.31%	0.19%
H	285	4.63%	2.85%

Singlets

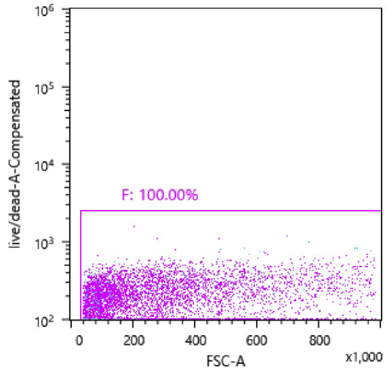

F

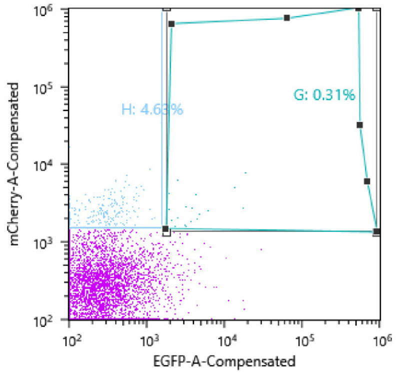

All Events

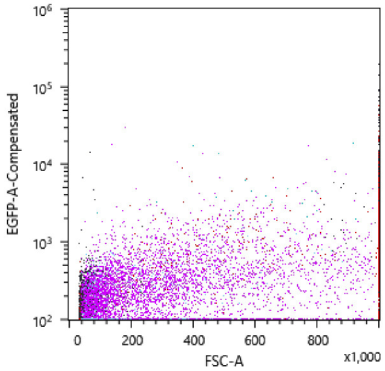

H

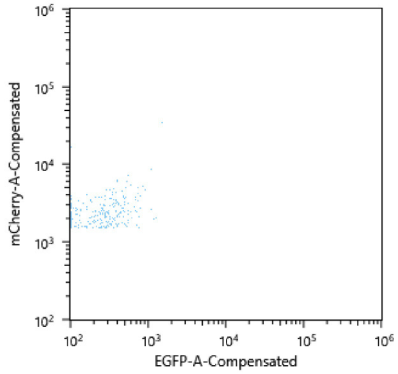

Gates and Statistics

Name	Events	%Parent	%Total
All Events	10,000	0.00%	100.00%
Scatter gate	6,891	68.91%	68.91%
Singlets	6,160	89.39%	61.60%
F	6,160	100.00%	61.60%
G	19	0.31%	0.19%
H	285	4.63%	2.85%

Sorting Result

Sorting Method	2 Way Tubes
Start Time	12/4/2018 2:35:24 PM
End Time	12/4/2018 2:36:06 PM

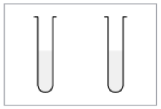

Collection Tube	Sort Gate	Sort Mode	Elapsed Time	Total Event	Target Ratio	Sorted Count	Sort Rate	Sort Efficiency	Aborted Count	Abort Rate
Left	H	Purity	00:00:41	138,740	6.25%	6,454	154.96eps	74.48%	2,211	53.09eps
Right	G	Purity	00:00:41	138,740	49.99%	58,395	1,402.07eps	84.20%	10,956	263.06eps
