# Supplementary material for: A zebrafish screen reveals Renin-angiotensin system inhibitors as neuroprotective via mitochondrial restoration in dopamine neurons
Source: eLife. 2021 Sep 22;10:e69795. doi: 10.7554/eLife.69795 (PMC8457844; doi:10.7554/eLife.69795)
Supplement: Figure 7—source data 4. [file elife-69795-fig7-data4.pdf]

PPMI Data and Publications Committee (DPC) review approval letter

David Bresnahan <dbresnahan@michaeljfox.org>

Thank you for submitting your manuscript entitled "A Zebrafish Screen Reveals Renin-Angiotensin System Inhibitors as Neuroprotective via Mitochondrial Restoration in Dopamine Neurons" to the PPMI Data and Publications Committee (DPC) for review. The DPC has reviewed your submission and have the following comments:

- No comments

The DPC did not have any other comments. Please consider this manuscript approved. We wish you luck with the publications process. Please email ppmi@michaeljfox.org with subject "Upcoming Publication from PPMI Analysis" when this manuscript has been accepted. Additionally, please send us a link to the article when it is published, as we would like to list it on our PPMI data publications website.

Best,
David

David Bresnahan
Research Cohorts Officer

THE MICHAEL J. FOX FOUNDATION FOR PARKINSON'S RESEARCH
Here. Until Parkinson's isn't.
Get involved at **MICHAELJFOX.ORG**
