# Supplementary material for: A zebrafish screen reveals Renin-angiotensin system inhibitors as neuroprotective via mitochondrial restoration in dopamine neurons
Source: eLife. 2021 Sep 22;10:e69795. doi: 10.7554/eLife.69795 (PMC8457844; doi:10.7554/eLife.69795)
Supplement: Supplementary file 1. [file elife-69795-supp1.docx]

**Supplemental File 1a.**

**Table S1. Primer designs for qPCR of RAAS pathway gene expression in DA neurons**

| Gene Name | Gene symbol | Function | Primer Sequences (5’->3’) | bp | Transcript ID |
| --- | --- | --- | --- | --- | --- |
| Angiotensin II receptor, type 1a | agtr1a | G protein-coupled receptor for angiotensin II | F: CTTCTCACTGCTCTCAGCGT  R: CATCCGTGGGACCCATTTCA | 163 | ENSDART00000021528.7 |
| Angiotensin II receptor, type 1b | agtr1b | G protein-coupled receptor for angiotensin II | F: TCCAGGGGTCTGTTGAGGAA  R: CAGTTTTCCACCACGCCAAG | 155 | ENSDART00000066834.5 |
| Angiotensin I converting enzyme | ace1 | Metallopeptidase involved in the conversion of angiotensin I to angiotensin II | F: CGCTCTACCTCAGCGTTCAT  R: GCCCACATGTTTCCAAGCAG | 115 | ENSDART00000114637.4 |
| Angiotensin I converting enzyme 2 | ace2 | Transmembrane protein catalyzing angiotensin II hydrolysis. Main entry point for coronavirus. | F: CTGATGCCTGTCTTCCAGCA  R: TTTCATCCCAACCCTGCTCC | 141 | ENSDART00000003712.8 |
| Angiotensinogen | agt | Precursor molecule for angiotensin I and the substrate for renin produced in the liver. | F: GGCTTCGACACCTCAAGGAA  R:ACACCACCTTGTTGAGTACCTTA | 192 | ENSDART00000010918.5 |
| Angiotensin II receptor, type 2 | at2 | G protein-coupled receptor. Regulation of aldosterone secretion | F: GTTCACGAACATCAGAACTCCC  R: TGAGGCTGTAAAAGGCAGGG | 187 | ENSDART00000051532.5 |
| Renin | ren | Enzyme cleaving angiotensinogen to form angiotensin I | F: CGGTGTACTACAGCAGGGAC  R: GTGTCGGTTGGGGCAGATAT | 160 | ENSDARG00000041858 |
| Prorenin receptor | atp6ap2 | Renin and prorenin receptor involved in the assembly of the ATPase protein pump | F: GTCTCTCTCAGCCGCAACAA  R: CTGCAACTGTAGCAAACGAACA | 184 | ENSDART00000178882.2 |
| Elongation factor 1-alpha | eef1a1 | Housekeeping gene; promote the GTP-dependent binding of aa-tRNA to the A-site of ribosomes | F: TTTTTGGGGTTTTGCAGGTCG  R: TTGGCCTTCTGGGCTTTCTG | 138 | ENSDART00000164533.2 |

**Supplemental File 1b.**

**Table S2. Differential expression of mitochondria-related genes for Olmesartan + CBE- or Olmesartan + MTZ-treated samples compared to controls.** 15 mitochondria-related genes with altered expression in CBE- and MTZ-treated samples are shown in the first column. The differential gene expression analysis was performed on CBE+olmesartan and MTZ+olmesartan samples against the control samples (α=0.05, FDR=0.1). Olmesartan treatment restores the expression of most genes back to normal. ns= not significant.

|  | CBE+olmesartan/control | MTZ+olmESartan/control |
| --- | --- | --- |
| Atp5l | ns (p-value; not significant) | 0.031 |
| Cox5aa | ns | ns |
| Cox6a1 | ns | ns |
| Cox6c | ns | 0.012 |
| Cox7a2a | ns | ns |
| Cox7c | ns | ns |
| Cox8a | 0.0083 | ns |
| Mrpl13 | 0.019 | ns |
| Mrps18c | ns | ns |
| Ndufa1 | 0.02484 | ns |
| Ndufb2 | ns | ns |
| Ndufs4 | ns | ns |
| Ndufv2 | ns | ns |
| Tomm6 | ns | ns |
| Trim3 | ns | ns |
