# Supplementary material for: A zebrafish screen reveals Renin-angiotensin system inhibitors as neuroprotective via mitochondrial restoration in dopamine neurons
Source: eLife. 2021 Sep 22;10:e69795. doi: 10.7554/eLife.69795 (PMC8457844; doi:10.7554/eLife.69795)
Supplement: Transparent reporting form [file elife-69795-transrepform1.docx]

***eLife’s* transparent reporting form**

We encourage authors to provide detailed information *within their submission* to facilitate the interpretation and replication of experiments. Authors can upload supporting documentation to indicate the use of appropriate reporting guidelines for health-related research (see [EQUATOR Network](http://www.equator-network.org/%20)), life science research (see the [BioSharing Information Resource](https://biosharing.org/)), or the [ARRIVE guidelines](http://www.plosbiology.org/article/info:doi/10.1371/journal.pbio.1000412) for reporting work involving animal research. Where applicable, authors should refer to any relevant reporting standards documents in this form.

If you have any questions, please consult our Journal Policies and/or contact us: [editorial@elifesciences.org](mailto:editorial@elifesciences.org).

**Sample-size estimation**

- You should state whether an appropriate sample size was computed when the study was being designed
- You should state the statistical method of sample size computation and any required assumptions
- If no explicit power analysis was used, you should describe how you decided what sample (replicate) size (number) to use

Please outline where this information can be found within the submission (e.g., sections or figure legends), or explain why this information doesn’t apply to your submission:

An a priori power analysis was not conducted to determine the sample size. The sample size was determined based on the efficient utilization of 96 well and 6 well plates, the size of the screening library, and on our previous work on establishing the screening model (Harrison et al. <https://doi.org/10.1371/journal.pone.0164645>). All experiments were carefully planned and conducted to ensure sufficient technical and biological replicates that allow for proper examination of data and statistical analysis.

The clinical data analysis of the PPMI data was a retrospective dataset thus sample size was not predetermined but rather all complete de novo PD patient data was included in the analysis along with a post hoc power analysis. Our analyzed data were reviewed and approved by the PPMI Data and Publications Committee.

**Replicates**

- You should report how often each experiment was performed
- You should include a definition of biological versus technical replication
- The data obtained should be provided and sufficient information should be provided to indicate the number of independent biological and/or technical replicates
- If you encountered any outliers, you should describe how these were handled
- Criteria for exclusion/inclusion of data should be clearly stated
- High-throughput sequence data should be uploaded before submission, with a private link for reviewers provided (these are available from both GEO and ArrayExpress)

Please outline where this information can be found within the submission (e.g., sections or figure legends), or explain why this information doesn’t apply to your submission:

The information on sample size and the replicates are all included in the figure legends along with the statistical tests used. All experiments include at minimum three biological and two technical replicates. No outliers were removed from the data. The 1mM captopril showed lethality to the larvae thus was not quantified which is stated in the figure legend.

**Statistical reporting**

- Statistical analysis methods should be described and justified
- Raw data should be presented in figures whenever informative to do so (typically when N per group is less than 10)
- For each experiment, you should identify the statistical tests used, exact values of N, definitions of center, methods of multiple test correction, and dispersion and precision measures (e.g., mean, median, SD, SEM, confidence intervals; and, for the major substantive results, a measure of effect size (e.g., Pearson's r, Cohen's d)
- Report exact p-values wherever possible alongside the summary statistics and 95% confidence intervals. These should be reported for all key questions and not only when the p-value is less than 0.05.

Please outline where this information can be found within the submission (e.g., sections or figure legends), or explain why this information doesn’t apply to your submission:

All the statistical tests, p-value, sample sizes, and methods are included in the figure legends and we also included a ‘statistical analysis’ section in the methods. The proper statistical test for the data normalization for the high throughput screening data and the PPMI clinical analysis were thoroughly consulted with the statisticians at the UC San Francisco Clinical and Translational Science Institute.

(For large datasets, or papers with a very large number of statistical tests, you may upload a single table file with tests, Ns, etc., with reference to sections in the manuscript.)

**Group allocation**

- Indicate how samples were allocated into experimental groups (in the case of clinical studies, please specify allocation to treatment method); if randomization was used, please also state if restricted randomization was applied
- Indicate if masking was used during group allocation, data collection and/or data analysis

Please outline where this information can be found within the submission (e.g., sections or figure legends), or explain why this information doesn’t apply to your submission:

Several components of the experiment including high throughput screening, image analysis, mass spectrometry of adult zebrafish brains, behavior studies were performed in a blinded manner and is stated in the manuscript. No specific randomization was performed but the larvae were separated into respective treatment groups upon birth from the same parents and our embedding technique utilizes both the before and after imaging which reduces variability as each sample serves as their own internal control.

Whilst larval zebrafish cannot be differentiated by gender, adult zebrafish samples were selected to best reflect gender balance in accordance with the NIH guidance when possible and stated in the manuscript.

**Additional data files (“source data”)**

- We encourage you to upload relevant additional data files, such as numerical data that are represented as a graph in a figure, or as a summary table
- Where provided, these should be in the most useful format, and they can be uploaded as “Source data” files linked to a main figure or table
- Include model definition files including the full list of parameters used
- Include code used for data analysis (e.g., R, MatLab)
- Avoid stating that data files are “available upon request”

Please indicate the figures or tables for which source data files have been provided:

A separate legend for the source data has been provided.
